# Low-quality of patient-reported outcome reporting in randomized clinical trials of major depressive disorder—a meta-epidemiological review

**DOI:** 10.3389/fpsyt.2023.1246938

**Published:** 2023-11-07

**Authors:** Jia Zhou, Han Qi, Jia Hu, Zizhao Feng, Gang Wang

**Affiliations:** ^1^Beijing Key Laboratory of Mental Disorders, National Clinical Research Center for Mental Disorders & National Center for Mental Disorders, Beijing Anding Hospital, Capital Medical University, Beijing, China; ^2^Advanced Innovation Center for Human Brain Protection, Capital Medical University, Beijing, China

**Keywords:** patient-reported outcome, major depressive disorder, randomized controlled trials, meta-epidemiological review, reporting quality evaluation

## Abstract

**Objective:**

Our goal was to review current peer-reviewed articles in which the BDI (Beck Depression Inventory), PHQ-9 (Patient Health Questionnaire), or QIDS-SR16 (16-Item Quick Inventory of Depressive Symptomatology) was used as the primary or secondary outcome measure and to evaluate the quality of PRO (Patient-Reported Outcome) reporting in RCTs (Randomized Controlled Trials) according to the 2013 PRO-specific CONSORT (Consolidated Standards of Reporting Trials) extension.

**Methods:**

We systematically searched in electronic databases. A study would be included if it included patients diagnosed with major depressive disorder according to the criteria of the Diagnostic and Statistical Manual of Mental Disorders (DSM) or International Classification of Diseases, version 10 (ICD-10) as participants, was a randomized controlled trial, included the BDI, PHQ-9, or QIDS-SR16 as the primary or secondary outcome measure, published between 1990 and 2013, and was in English. Two of the authors evaluated the quality of PRO reporting according to the 2013 CONSORT-PRO. Logistic regression were used to evaluate the association between reporting completeness and trial characteristics.

**Results:**

A total of 116 studies were included. These studies were conducted in 25 countries. Sample sizes ranged from 12 to 750. The CONSORT-PRO was not cited in any one of the included studies. Among the 116 studies, 2 (1.72%) studies introduced the rationale for PRO assessment, 60 (51.72%) studies explicitly stated statistical approaches for dealing with missing data, 87 (75.00%) studies reported PRO outcome data at baseline and at subsequent time points. The mean score of reporting completeness was 66.24%. Significantly higher reporting completeness was found for RCTs published after 2013 (OR, 95%CI: 3.81, 1.32–10.99). Studies with a higher sample size were more completely reported than studies with a lower sample size (OR, 95%CI: 1.01, 1.00–1.02).

**Conclusion:**

The CONSORT-PRO guidance was rarely cited. The quality of PRO reporting in depression studies requires improvement. This result may be meaningful for the promotion of PRO reporting in RCTs.

## 1. Introduction

Patient-reported outcome (PRO), as defined by the US Food and Drug Administration (FDA), is “a measurement of a patient’s health condition that is reported directly by the patient” (1). PRO is increasingly recognized by regulators, clinicians, and patients as a valuable tool to measure treatment benefits in terms of the alleviation of the patients’ symptoms and improvement of their pertinent function (2, 3). Responding to this imperative, PRO endpoints are more commonly incorporated in clinical trial design (4) as the primary or secondary outcome measures (5). Despite this, international reviews indicated that PRO are still underutilized (6). Furthermore, the quality of PRO content in many reports is often suboptimal (7, 8).

To make PRO data from randomized controlled trials (RCTs) meaningful, it is critical to have the study and PRO designed well, analyzed appropriately, and reported in a way that makes the results accessible and useful for the critical appraisal of the study results (9). To address this need, corresponding recommendations have been developed, such as Standard Protocol Items: Recommendations for Interventional Trials-PRO extension (SPIRIT-PRO) (10), Setting International Standards in Analyzing Patient-Reported Outcomes and Quality of Life Endpoints Data (SISAQOL) (11), Consolidated Standards of Reporting Trials Statement-PRO extension (CONSORT-PRO) (12), and the COSMIN reporting guideline for studies on measurement properties of patient-reported outcome measures (13). All these guidelines provided good references for good methodological practices that can meaningfully and reliably inform patient safety, treatment choices, and policy decisions through PRO. However, the implementation of these recommendations in RCTs remains suboptimal (14). For instance, a literature review focused on PRO reporting in RCTs evaluating systemic cancer therapy and found that the quality of the reporting was rather low: only 26% of RCTs included a description of the prespecified PRO hypothesis, only 16% of RCTs included methods for PRO data collection, and only 37% of RCTs introduced the statistical approaches for managing missing data (15).

Major depressive disorder (MDD) has been ranked as one of the leading causes of disability worldwide and is projected to cause the heaviest burden by 2030 (16). It is a debilitating disease characterized by depressed mood, diminished interests or pleasure, impaired cognitive function, disturbed sleep or appetite and suicidal ideation (17). MDD is primarily a subjective experience, and the degree of impairment was directly related to symptom severity. Therefore, PROs are increasingly utilized as essential endpoints for clinical studies (18) and may provide clinically important information not accessible through clinician rating scales (19). However, according to a review conducted by Minley et al., the completeness of reporting PROs in RCTs addressing MDD was inadequate. A total of 49 RCTs published between 2016 and 2020 were identified, and the overall mean completion percent for the CONSORT-PRO checklist adaptation was 56.74% (20). The Beck Depression Inventory (BDI), Patient Health Questionnaire-9 (PHQ-9) and Quick Inventory of Depressive Inventory (QIDS-SR16) are frequently used self-report instruments in clinical trials of major depressive disorder (20–22). However, there is limited data regarding the quality of PRO reporting in RCTs of MDD before and after the publish of 2013 PROs-specific CONSORT extension.

Responding to this problem, our goal was to review current peer-reviewed articles in which the BDI, PHQ-9, or QIDS-SR16 was used as the primary or secondary outcome measure and to evaluate the quality of PRO reporting in RCTs according to the 2013 PROs-specific CONSORT extension. By doing so, we hope to comprehensively evaluate the current condition of PRO reporting and explore the impact of PRO-specific CONSORT extension on report quality.

## 2. Methods

### 2.1. Study selection

In June 2020, we systematically searched in electronic databases including the Cochrane Library, PubMed, Embase, and Web of Science for articles published in English from January 1990 to June 2020. In September 2023, an update of the literature search was conducted. The search started from 1990, since ICH’s inception in 1990. Then the ICH process has gradually evolved, which symbolizes progress in the development of guidelines on safety, quality and efficacy topics. The search strategy and associated terms were based on the inclusion and exclusion criteria for the patient population, outcomes, and study design: (depress*[title]) and ((“9 item patient health questionnaire”[title/abstract]) or (“nine item patient health questionnaire”[title/abstract]) or (“patient health questionnaire 9”[title/abstract]) or (“phq-9”[title/abstract]) or (“quick inventory of depressive symptomatology self-report”[title/abstract]) or (“qids-sr”[title/abstract]) or (“beck depression inventory”[title/abstract]) or (“bdi”[title/abstract])) and ((randomized controlled trial [pt] or controlled clinical trial [pt] or randomized [tiab] or placebo [tiab] or clinical trials as topic [mesh: noexp] or randomly [tiab] or trial [ti]) not (animals[mh] not humans[mh])) and (1990:2023[pdat]).

A study would be included if it included patients diagnosed with major depressive disorder according to the criteria of the Diagnostic and Statistical Manual of Mental Disorders (DSM) or International Classification of Diseases, version 10 (ICD-10) as participants, was a randomized controlled trial, included the BDI (Beck Depression Inventory), PHQ-9 (Patient Health Questionnaire), or QIDS-SR16 (16-Item Quick Inventory of Depressive Symptomatology) as the primary or secondary outcome measure, and was in English. A study would be excluded if the full text was unavailable or it is a secondary analysis of RCT. Studies of comorbid MDD in other diseases were not excluded. Two of the authors (JH and HQ) independently screened articles by titles and abstracts and reviewed the full texts of selected articles, any disagreement in the literature selection process was resolved by a consensus and/or a discussion with a senior investigator (JZ).

### 2.2. Scoring CONSORT-PRO

According to 2013 CONSORT-PRO, there were 52 entries evaluated. The scoring methodology was adapted from Mercieca-Bebber et al. (23) and Minley et al. (20). Item 3b (important changes to methods after trial commencement (such as eligibility criteria), with reasons), 6b (any changes to trial outcomes after the trial commenced, with reasons), and 14b (why the trial ended or was stopped) of CONSORT-PRO were excluded from scoring as it was difficult to verify without checking the trial protocols. Adherence to these items would be only described using frequency of adequately reported (Table 1). Conditional entries are not included in scoring, including 7b (When applicable, explanation of any interim analyses and stopping guidelines), 11a (If done, who was blinded after assignment to interventions (for example, participants, care providers, those assessing outcomes) and how), and 17b (For binary outcomes, presentation of both absolute and relative effect sizes is recommended). Furthermore, assessment of item 7a was dependent on whether PRO was the primary endpoint for RCTs. Each of the other 45 items was weighted with equal importance. Each item was recorded “yes” and scored 1 if it was adequately reported. The item was labeled “no” and scored 0 if it was not comprehensively reported or not reported at all. The maximum score of RCTs was 45. Reporting score of RCTs was calculated by adding all the items score and dividing by the possible maximum score.

**Table 1:** Quality of PROs reporting, rated using items of the 2013 extensions of the CONSORT statement (*N* = 116).

Item	Descriptor of the 2010 CONSORT criteria	Trials		Item	Descriptor of the 2013 PRO-specific extension or elaboration	Trials	
		*n*	%			*n*	%
**Title and abstract**							
1a	Identification as a randomized trial in the title	93	80.17				
1b	Structured summary of trial design, methods, results, and conclusions	96	82.76	P1b	Identification of the PROs in the abstract as a primary or secondary outcome	103	88.79
**Introduction**							
2a	Scientific background and explanation of rationale	116	100	P2a	Including background and rationale for PRO assessment	2	1.72
2b	Specific objectives or hypotheses	114	98.28	P2b	The PRO hypothesis should be stated	5	4.31
					Relevant domains identified, if applicable	1	0.86
**Methods**							
3a	Description of trial design (such as parallel, factorial), including allocation ratio	102	87.93				
3b	Important changes to methods after trial commencement (such as eligibility criteria), with reasons	16	13.79				
4a	Eligibility criteria for participants	115	99.14				
4b	Settings and locations where the data were collected	106	91.38				
5	The interventions for each group with sufficient details to allow replication, including how and when they were actually administered	115	99.14				
6a	Completely defined prespecified primary and secondary outcome measures, including how and when they were assessed	103	88.79	P6a	Reference of the PROs instrument	102	87.93
					Statement of the person completing the PROs	88	75.86
					Methods of data collection (paper, telephone, electronic, other)	15	12.93
6b	Any changes to trial outcomes after the trial commenced, with reasons	4	3.45				
7a	How sample size was determined	50	43.10				
7b	When applicable, explanation of any interim analyses and stopping guidelines	3	2.59				
**Randomization**							
8a	Method used to generate the random allocation sequence	73	62.93				
8b	Type of randomization; details of any restriction (such as blocking and block size)	57	49.14				
9	Mechanism used to implement the random allocation sequence (such as sequentially numbered containers), describing any steps taken to conceal the sequence until interventions were assigned	66	56.90				
10	Who generated the random allocation sequence, who enrolled participants, and who assigned participants to interventions	51	43.97				
11a	If done, who was blinded after assignment to interventions (for example, participants, care providers, those assessing outcomes) and how	70	60.34				
11b	If relevant, description of the similarity of interventions	34	29.31				
12a	Statistical methods used to compare groups for primary and secondary outcomes	111	95.69	P12a	Statistical approaches for dealing with missing data are explicitly stated	60	51.72
12b	Methods for additional analyses, such as subgroup analyses and adjusted analyses	78	67.24				
**Results**							
13a	For each group, the numbers of participants who were randomly assigned, received intended treatment, and were analyzed for the primary outcome	114	98.28	P13a	The number of PRO outcome data at baseline and at subsequent time points should be made transparent	87	75.00
13b	For each group, losses and exclusions after randomization, together with reasons	98	84.48				
14a	Dates defining the periods of recruitment and follow-up	75	64.66				
14b	Why the trial ended or was stopped	3	2.59				
15	A table showing baseline demographic and clinical characteristics for each group	106	91.38	P15	Including baseline PRO data when collected	96	82.76
16	For each group, number of participants (denominator) included in each analysis and whether the analysis was by original assigned groups	106	91.38	P16	Required for PRO results	101	87.07
17a	For each primary and secondary outcome, results for each group, the estimated effect size, and its precision (such as 95% confidence interval)	64	55.17	P17a	For multidimensional PRO results from each domain and time point	0	0
17b	For binary outcomes, presentation of both absolute and relative effect sizes is recommended	18/21	85.71				
18	Results of any other analyses performed, including subgroup analyses and adjusted analyses, distinguishing prespecified from exploratory	69	59.48	P18	Including PRO analyses, where relevant	65	56.03
19	All-important harms or unintended effects in each group (for specific guidance see CONSORT for harms)	62	53.35				
**Discussion**							
20	Trial limitations, addressing sources of potential bias, imprecision, and, if relevant, multiplicity of analyses	104	89.66	P20/21	PRO-specific limitations and implications for generalizability and clinical practice	22	18.97
21	Generalizability (external validity, applicability) of the trial findings	79	68.10				
22	Interpretation consistent with results, balancing benefits and harms, and considering other relevant evidence	116	100	P22	PRO data should be interpreted in relation to clinical outcomes including survival data, where relevant	19	16.38
**Other information**							
23	Registration number and name of trial registry	69	59.48				
24	Where the full trial protocol can be accessed, if available	59	50.86				
25	Sources of funding and other support (such as supply of drugs), role of funders	97	83.62				

CONSORT, Consolidated Standards of Reporting Trials; PRO, Patient-Reported Outcome.

Moreover, according to the CONSORT-PRO scores, studies were categorized into “moderate to good,” or “poor” reporting according to pre-specified thresholds. The RCT was recorded to be “moderate to good” if it addressed more than 60% of the CONSORT-PRO items, and “poor” if ≤60%.

### 2.3. Trial characteristics and quantitative systems

Characteristics of the trials, such as the year of publication, country, single/multicenter, number of groups, intervention, and sample size, were collected. Two of the authors (JH and HQ) evaluated the quality of PROs reporting according to the 2013 CONSORT-PRO. These two authors examined each article independently. If there was uncertainty in the understanding of an article, the third author (JZ) would resolve it through consensus evaluation. If a PRO was clearly determined as a primary outcome, it would be labeled as primary outcome, otherwise it was considered a secondary outcome. The CONSORT-PRO was published in 2013, a stratified description (1990 to 2012 or 2013 to 2020) of the key evaluations was conducted.

### 2.4. Statistical analysis

We reported our search results and the frequency of each trial characteristic of the RCTs. Additionally, we reported the frequency of RCTs that cite CONSORT-PRO. Next, we reported the frequency of each CONSORT-PRO item in all RCTs. To determine significant differences between different groups, we used χ^2^ tests for categorical variables and Wilcoxon Signed Rank Test for continuous variables, respectively. Tests were 2-sided at the 0.05 significance level. Bivariate logistic regression was applied to estimate the odds ratios (ORs) and 95% confidence interval (CI) for associations between study characteristics and quality of PRO reporting.

## 3. Results

### 3.1. Characteristics of selected randomized controlled trials

**Figure 1:**
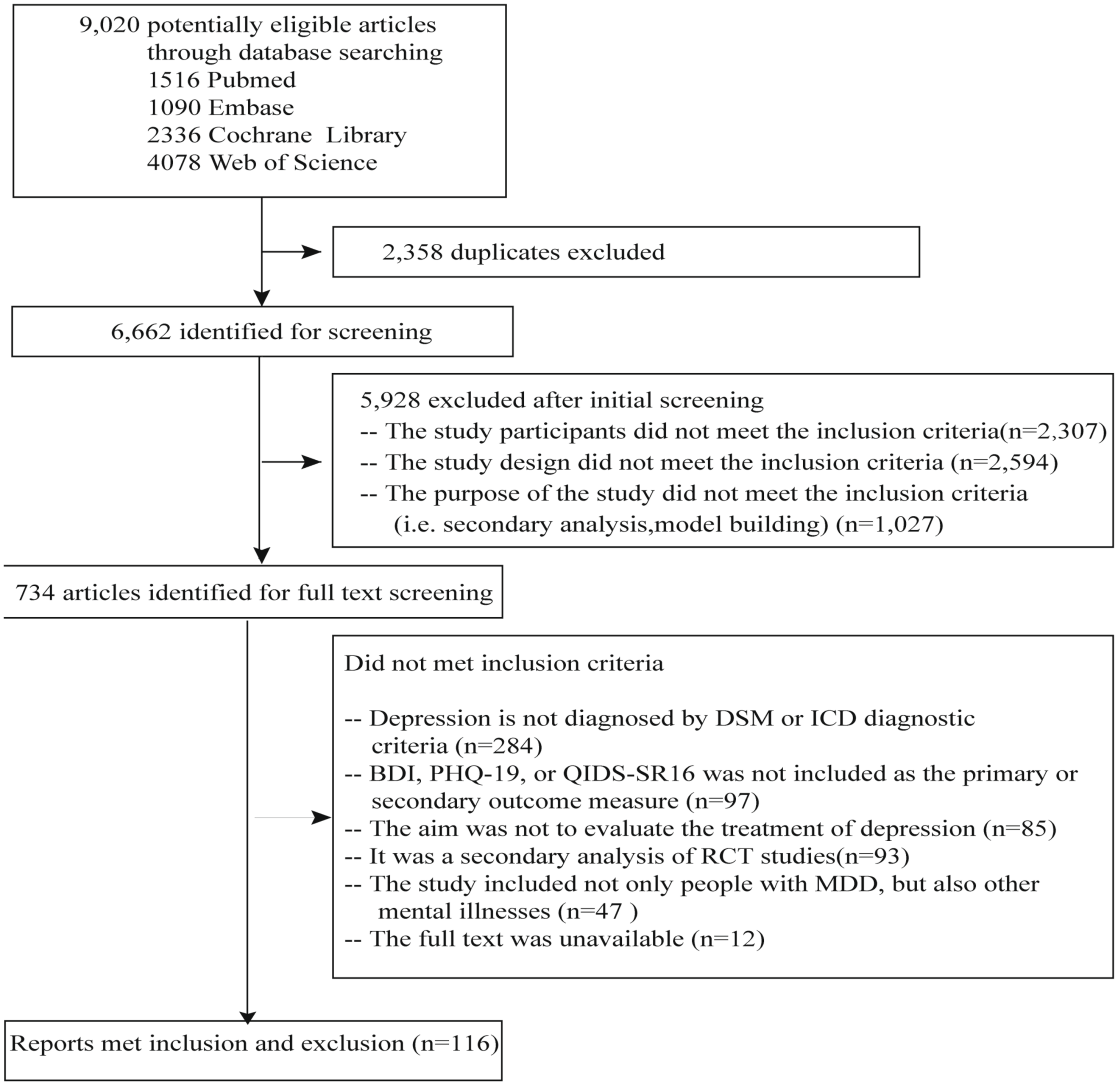
Flow chart.

In total, 9,020 studies were found through the database search of studies published from January 1990 to June 2023 (Figure 1). After excluding the duplicates, we screened the articles and excluded the articles without full text or the studies in which the participants were not diagnosed with major depressive disorder; in the end, 116 studies remained (Figure 1). Among them, 31 (26.72%) were published between 1990 and 2012, and 85 (73.28%) were published between 2013 and 2023. These studies were conducted in 25 countries. Of these studies, 74 (73.79%) were single-center and 42 (36.21%) were multicenter. The criteria of the Diagnostic and Statistical Manual of Mental Disorders (DSM) were used in 92 studies, and the ICD-10 criteria were used in 24 (20.69%) studies. Among all the studies, 87 (75.00%) were two-arm, and 27 (23.28%) were three-arm. The intervention in 45 (38.79%) of the studies was psychotherapy, in 22 (18.97%) was physiotherapy, in 37 (31.90%) was pharmacotherapy, and in 12 (10.34%) were other treatments (e.g., supportive text messages, expressive writing, and measurement-based care). Sample sizes ranged from 12 to 750. The QIDS-SR16 was used as primary or secondary outcomes in eight studies, PHQ-9 was used in eight studies, and BDI was used in 100 (86.21%) studies. Among these studies, patient-reported outcomes (i.e., PHQ-9, QIDS-SR16, and BDI) were included as primary outcomes in 73 (62.93%) and secondary outcomes in 43 (37.07%), respectively (Table 2).

**Table 2:** Characteristics of the studies.

Study characteristics	Trials	
	*n*	%
**Year of report**		
1990 to 2012	31	26.72
2013 to 2023	85	73.28
**Site**		
Single-center	74	63.79
Multi-center	42	36.21
**Diagnostic criteria for MDD**		
DSM	92	79.31
ICD-10	24	20.69
**Intervention**		
Psychotherapy	45	38.79
Physiotherapy	22	18.97
Pharmacotherapy	37	31.90
Other treatments	12	10.34
**Outcomes**		
Primary outcome	73	62.93
Secondary outcome	43	37.07
**Measurement**		
BDI	100	86.21
PHQ-9	8	6.90
QIDS-SR16	8	6.90

MDD, major depressive disorder; DSM, The diagnostic and statistical manual of mental disorders; ICD-10, International Classification of Diseases, version 10; BDI, Beck Depression Inventory; PHQ-9, Patient Health Questionnaire; QIDS-SR16, The 16-Item Quick Inventory of Depressive Symptomatology (Self-Report Versions).

### 3.2. Overall quality of PROs reporting

The mean score of reporting was 66.24%. The CONSORT-PRO was not cited in any one of the included studies published after 2013. Among the 116 studies, 2 (1.72%) studies introduced the rationale for PRO assessment, 102 (89.93%) studies included reference of the PROs instrument, 60 (51.72%) studies explicitly stated statistical approaches for dealing with missing data, 87 (75.00%) studies reported PRO outcome data at baseline and at subsequent time points, and 22 (18.97%) studies discussed PRO-specific limitations and implications for generalizability and clinical practice (Table 1).

### 3.3. Quality of PROs reporting before and after the release of CONSORT-PRO

Among the 116 studies, 31 (26.72%) were published between 1990 and 2012, and 85 (73.28%) were published between 2013 and 2023. Their mean score of reporting was 58.78 and 68.97%, respectively. Significant improvement of reporting completeness was seen in P12a (statistical approaches for dealing with missing data are explicitly stated), P15 (Including baseline PRO data when collected), and P22 (PRO data should be interpreted in relation to clinical outcomes, including survival data, where relevant). Detailed information can be seen in Table 3.

**Table 3:** Quality of PROs reporting before and after the release of CONSORT-PRO (*N* = 116).

Descriptor of the 2013 PRO-specific extension or elaboration	1990–2012, *n* (%)	2013–2023, *n* (%)	χ^2^	*p*
P1b: Identification of the PROs in the abstract as a primary or secondary outcome	27 (87.10)	76 (89.41)	0.12	0.73
P2a: Including background and rationale for PRO assessment	1 (3.23)	1 (1.18)	0.56	0.45
P2b: The PRO hypothesis should be stated	2 (6.45)	3 (3.53)	0.47	0.49
P2b: Relevant domains identified, if applicable	0 (0.00)	1 (1.18)	0.37	0.54
P6a: Reference of the PROs instrument	26 (83.87)	76 (89.41)	0.66	0.42
P6a: Statement of the person completing the PROs	22 (70.97)	66 (77.65)	0.55	0.46
P6a: Methods of data collection (paper, telephone, electronic, other)	4 (12.90)	11 (12.94)	0.00	0.99
P12a: Statistical approaches for dealing with missing data are explicitly stated	9 (29.03)	51 (60.00)	8.72	<0.01^*^
P13a: The number of PRO outcome data at baseline and at subsequent time points should be made transparent	21 (67.74)	66 (77.65)	1.19	0.28
P15: Including baseline PRO data when collected	21 (67.74)	75 (88.24)	6.69	<0.01^*^
P16: Required for PRO results	27 (87.10)	74 (87.06)	0.00	0.99
P17a: For multidimensional PRO results from each domain and time point	0	0	–	–
P18: Including PRO analyses, where relevant	14 (45.16)	51 (60.00)	2.03	0.15
P20/21: PRO-specific limitations and implications for generalizability and clinical practice	8 (25.81)	14 (16.47)	1.29	0.26
P22: PRO data should be interpreted in relation to clinical outcomes including survival data, where relevant	1 (3.23)	18 (21.18)	5.34	0.02^*^

CONSORT, Consolidated Standards of Reporting Trials; PRO, Patient-Reported Outcome.

### 3.4. The associations between study characteristics and quality of PRO reporting

Among the 116 studies, 41 (35.34%) were recorded to be “poor,” and 75 (64.55%) recorded to be “moderate to good.” Our bivariate regression analyses revealed that RCTs published after 2013 were more completely reported than RCTs published between 1990 and 2012 (OR, 95%CI: 3.81, 1.32–10.99). Studies with a higher sample size were more completely reported than studies with a lower sample size (OR, 95%CI: 1.01, 1.00–1.02). Further results of these analyses can be found in Tables 4 5.

**Table 4:** Differences of characteristics between RCTs at different scoring levels.

Variables	Poor, *n* (%)	Moderate to good, *n* (%)	χ^2^	*p*
Year of publish			9.56	<0.01^*^
1990–2012	18 (58.06%)	13 (41.94%)		
2013–2023	23 (27.06%)	62 (72.94%)		
Study site			3.83	0.05
Single-site	31 (41.89%)	43 (58.11%)		
Multi-site	10 (23.81%)	32 (76.19%)		
Diagnostic criteria			0.06	0.80
DSM	32 (34.78%)	60 (65.22%)		
ICD	9 (37.50%)	15 (62.50%)		
Intervention			8.51	0.04^*^
Physiotherapy	12 (54.55%)	10 (45.45%)		
Psychotherapy	10 (22.22%)	35 (77.78%)		
Pharmacotherapy	16 (43.24%)	21 (56.76%)		
Other treatments	3 (25.00%)	9 (75.00%)		
Scale of PRO			4.24	0.12
BDI	39 (39.00%)	61 (61.00%)		
PHQ-9	1 (12.50%)	7 (87.50%)		
QIDS-SR16	1 (12.50%)	7 (87.50%)		
Endpoint			2.334	0.13
Primary	19 (44.19%)	24 (55.81%)		
Secondary	22 (30.14%)	51 (69.86%)		
Sample size	55.00 (35.00–91.00)^#^	94.00 (47.00–200.00)^#^	−3.58	<0.01^*^

BDI, Beck Depression Inventory; PHQ-9, Patient Health Questionnaire; QIDS-SR16, 16-Item Quick Inventory of Depressive Symptomatology; DSM, Diagnostic and Statistical Manual of Mental Disorders; ICD, International Classification of Diseases; PRO, Patient-Reported Outcome. # indicates median (Inter-Quartile Range).

**Table 5:** Bivariate regression analyses between study characteristics and quality of PRO reporting.

Parameter	Estimate	Standard error	Wald χ^2^	*p*	OR, 95%CI
Intercept	−0.8284	0.9093	0.8300	0.3623	
Year of publish	1.3375	0.5406	6.1223	0.0133	3.81, 1.32–10.99^*^
Study site	0.1966	0.5809	0.1146	0.7350	1.21, 0.39–3.80
Diagnostic criteria	−0.4653	0.6353	0.5364	0.4639	0.63, 0.18–2.18
**Intervention (ref. = other treatments)**					
Physiotherapy	−0.7726	0.8620	0.8034	0.3701	0.46, 0.09–2.50
Psychotherapy	0.0914	0.8579	0.0113	0.9152	1.10, 0.20–5.89
Pharmacotherapy	−0.8641	0.8246	1.0980	0.2947	0.42, 0.08–2.12
**Scale (ref. = BDI)**					
PHQ-9	0.9398	1.1691	0.6463	0.4214	2.56, 0.26–25.31
QIDS-SR16	0.9750	1.2170	0.6419	0.4230	2.65, 0.24–28.79
Endpoint	0.0255	0.5000	0.0026	0.9593	1.03, 0.39–2.73
Sample size	0.00799	0.00332	5.7977	0.0160	1.01, 1.00–1.02^*^

BDI, Beck Depression Inventory; PHQ-9, Patient Health Questionnaire; QIDS-SR16, 16-Item Quick Inventory of Depressive Symptomatology; DSM, Diagnostic and Statistical Manual of Mental Disorders; ICD, International Classification of Diseases.

## 4. Discussion

Our study highlighted that the quality of PRO reporting in depression studies requires improvement, even though significant improvement was seen after the release of CONSORT-PRO. This result can provide guidance for reporting information in future studies.

The CONSORT-PRO was published in 2013 and provided an evidence-based list of items recommended for inclusion in trial reports. The CONSORT-PRO facilitates more complete and transparent reporting (23), but it was not cited in any of the included studies published after 2013. A review of the reporting of patient-reported outcomes in elderly patients with hip fractures found that no study has mentioned the CONSORT-PRO or any other PRO-reporting guidelines (24). Between February 2013 and 17 December 2015, only 26 RCTs cited the CONSORT-PRO appropriately, representing a minute proportion of RCTs that reported PRO results during that period (23). A review of randomized controlled trials of hematological malignancies reported a similar finding: only 6% (*n* = 4) of 71 included studies cited the CONSORT-PRO extension explicitly (25). A review of cystic fibrosis randomized controlled trials also found inadequate reporting of patient-reported outcomes using CONSORT-PRO (26). Fifty-nine eligible RCTs were included, and their mean completeness of reporting was 38.38%. There are some potential barriers to citing the CONSORT-PRO, such as lacking endorsement from journals and a widespread lack of awareness of its existence and/or importance. It would be ideal if the journals would set requirements of a reference to the CONSORT-PRO, in order to facilitate more scientific reports and reduce research waste. A failure to cite the CONSORT-PRO may not imply a failure to use it, but it does imply that the extent of awareness remains unsatisfactory overall. We recommend referring to these international criteria in future RCT publications that include PRO data.

Furthermore, we found that the mean score of reporting was 66.24%, which means the overall reporting of PROs was suboptimal in current RCTs of depression. Similar to our result, a review from Minley et al. reported a mean CONSORT-PRO completion score of 56.7%, and also found that training on the application of PRO data in studies of MDD is needed (20). In our review, 5 (4.31%) RCTs report a PRO hypothesis or relevant PRO domains, lower than previous reviews of trials of ovarian cancer (19%) (27). Because PRO data are usually collected at multiple time points, a lack of clear hypotheses may obstruct the accurate evaluation of statistical analyses and research results. Therefore, in the design stage of a study, it is important to establish a PRO-related study hypothesis, specify the interested PRO-related domains, and plan for statistical analysis. Other important PRO criteria that were rarely met include the “interpretation of PRO findings” which was met in only about 14% of all cases. Failure to adequately interpret results can lead to unwarranted conclusions and limit the objectiveness of medical research. PRO-specific limitations and implications for generalizability and clinical practice were well reported in 18.97% of all RCTs of depression included in our study. This proportion was similar to that of the studies on multiple myeloma examined in another review (28) but was much lower than that of the 71 RCTs of hematological malignancies (25). More efforts should be made to improve the quality of reporting because it is helpful for clinicians and patients to assess treatment tolerability and make therapeutic decisions.

Importantly, as an integral part of PRO analysis and interpretation, handling missing data is inevitably, but about half of the articles included in this study did not state their statistical approaches for dealing with missing data completely. A systematic review assessing PRO reporting in studies on multiple myeloma found that only 23.0% reported a statistical plan for handling missing data (28). In another review of PRO reporting in randomized controlled trials of hematological malignancies, the proportion of missing data was reported in 51 (72%) of the 71 RCTs, but approaches used to handle missing data were described in only 26 (37%) trials (25). A review of RCTs on breast cancer published in 2018 indicated that the information about how the missing data were handled was omitted in 48 (73%) studies (29). Similarly, another review of 557 RCTs on cancer showed that the statistical approaches used to deal with missing PRO data was reported in only 20% studies (30). It is commonly known that missing data are common, and sometimes unavoidable, and it can potentially lead to loss of information, biased estimates, and impaired power and interpretability (31). Sensitivity analyses is important to verify the stability and robustness of the findings. Furthermore, transparent reporting of the missing data at each time point, is important for the assessment of potential bias in the PRO results. While deficiencies in reporting were common and there is room for improvement.

In our study, the need for clear, and comprehensive PRO-specific reporting to standardize PRO methodology, improving PRO data quality and minimizing the potential for bias is reinforced. Nonetheless, our study still has limitations. First, we reviewed published articles but not study protocols. Some criteria of the CONSORT-PRO may have been reported in the study protocol. So, our findings should not be interpreted as an appraisal of the overall quality of all PRO studies. However, CONSORT-PRO advised that its criteria should not only be addressed in the study protocol, but also be addressed in the final report. Second, despite our thorough search strategy, our analysis was limited to published studies. Also, only the studies in which BDI, PHQ-19, or QIDS-SR16 was included as the primary or secondary outcome measure were analyzed, and RCTs with other PRO endpoints have been missed. Third, the factors influencing the quality of reporting in the analysis may be not comprehensive, inclusion of more comprehensive factors is needed in future. Despite these limitations, our data may serve as a benchmark to monitor the quality of PRO reporting in future depression studies. It provides a broad overview of the quality of PRO reporting in RCT on MDD and reveals the impact of study characteristic and CONSORT-PRO on report quality.

## 5. Conclusion

The significant improvement in PRO-reporting was seen after the release of CONSORT-PRO. The quality of PRO reporting in depression studies still requires improvement. More efforts should be made to promote adequate reporting. We believe that increasing the application of the CONSORT-PRO in studies and the endorsement of CONSORT-PRO guidelines by the journals may be meaningful for the promotion of PRO reporting in RCTs.

## Data availability statement

The original contributions presented in the study are included in the article/Supplementary material, further inquiries can be directed to the corresponding author.

## Author contributions

GW: conceptualization, review and editing. JZ, HQ, and JH methodology and formal analysis. JZ: writing—original draft preparation. ZF: review and language editing. All authors contributed to the article and approved the submitted version.
